# ZF-CxxC domain-containing proteins, CpG islands and the chromatin connection

**DOI:** 10.1042/BST20130028

**Published:** 2013-05-23

**Authors:** Hannah K. Long, Neil P. Blackledge, Robert J. Klose

**Affiliations:** *Department of Biochemistry, University of Oxford, South Parks Road, Oxford OX1 3QU, U.K.; †Weatherall Institute of Molecular Medicine, University of Oxford, John Radcliffe Hospital, Oxford OX3 9DS, U.K.

**Keywords:** chromatin, CpG island, DNA demethylation, DNA methylation, epigenetics, transcription, AF9, ALL1–fused gene from chromosome 9 protein, ASH2L, absent, small or homeotic 2-like, BAH, bromo-adjacent homology, CFP1, CxxC finger protein 1, CGBP, CpG-binding protein, CGI, CpG island, ChIP-seq, chromatin immunoprecipitation sequencing, DNMT1, DNA methyltransferase 1, DPY-30, dosage compensation protein 30, ENL, eleven-nineteen leukaemia, ESC, embryonic stem cell, FBXL19, F-box and leucine-rich repeat protein 19, HDAC, histone deacetylase, 5hmC, 5-hydroxymethylcytosine, IDAX, inhibition of the Dvl and axin complex protein, JmjC, Jumonji C, KDM, lysine demethylase, MBD, methyl-CpG-binding domain, 5mC, 5-methylcytosine, MLL, mixed lineage leukaemia protein, PRC, polycomb group repressive complex, PHD, plant homeodomain, RbBP5, retinoblastoma-binding protein 5, RFTS, replication foci-targeting sequence, RING, really interesting new gene, RNAPII, RNA polymerase II, SEC, super-elongation complex, SETD1, SET domain 1, shRNA, short hairpin RNA, TET, ten-eleven translocation, WDR, WD40 repeat, YY1, Yin and Yang 1, ZF-CxxC, zinc finger-CxxC

## Abstract

Vertebrate DNA can be chemically modified by methylation of the 5 position of the cytosine base in the context of CpG dinucleotides. This modification creates a binding site for MBD (methyl-CpG-binding domain) proteins which target chromatin-modifying activities that are thought to contribute to transcriptional repression and maintain heterochromatic regions of the genome. In contrast with DNA methylation, which is found broadly across vertebrate genomes, non-methylated DNA is concentrated in regions known as CGIs (CpG islands). Recently, a family of proteins which encode a ZF-CxxC (zinc finger-CxxC) domain have been shown to specifically recognize non-methylated DNA and recruit chromatin-modifying activities to CGI elements. For example, CFP1 (CxxC finger protein 1), MLL (mixed lineage leukaemia protein), KDM (lysine demethylase) 2A and KDM2B regulate lysine methylation on histone tails, whereas TET (ten-eleven translocation) 1 and TET3 hydroxylate methylated cytosine bases. In the present review, we discuss the most recent advances in our understanding of how ZF-CxxC domain-containing proteins recognize non-methylated DNA and describe their role in chromatin modification at CGIs.

## Background

The vast majority of cytosine methylation in vertebrates is found within the context of cytosine guanine dinucleotides (CpGs), occurring in up to 80% of CpGs in the genome [1,2]. Methylated CpGs are found broadly across the genome, covering both genic and intergenic regions and are specifically recognized by proteins that encode MBDs (methyl-CpG-binding domains) [3,4]. MBD proteins are generally found associated with co-repressor complexes and are thought to impose a repressive chromatin state through the activity of HDACs (histone deacetylases) [5]. In some instances, methylation of CpGs can also block access of transcription factors to their cognate binding sites to counteract transcription [5–7].

Despite the prevalence of CpG methylation, short (~1–2 kb) contiguous CpG-rich stretches of the genome exist which are generally refractory to DNA methylation [8,9]. These regions are known as CGIs (CpG islands) and are found in approximately 50–70% of vertebrate gene promoters suggesting they may play a role in gene regulation [2,10,11]. However, the precise mechanisms by which CGIs contribute to gene expression have remained largely enigmatic.

**Figure 1:**
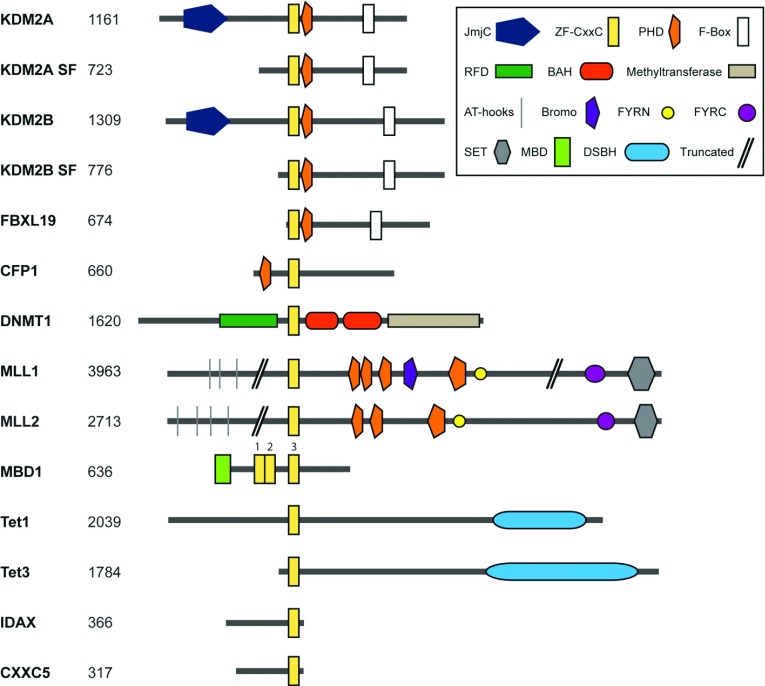
A family of ZF-CxxC domain-containing proteins An illustration of the domain architecture of all 12 mouse ZF-CxxC domain-containing proteins. The proteins are drawn to scale with the number of amino acids in the protein indicated on the left. The proteins are shown with the N-terminus on the left and all proteins are centred at the ZF-CxxC domain. In the case of KDM2A and KDM2B, alternative downstream promoters give rise to short forms of each protein (SF). For MBD1, the three ZF-CxxC domains are numbered 1–3 and the protein was aligned using the third ZF-CxxC domain with non-methylated CpG DNA-binding activity [95,96]. Domain annotation was performed using a sequence search from the Pfam database (http://pfam.sanger.ac.uk/). All sequences, apart from for TET3, were taken from NCBI. All sequences are from mouse except where stated. NCBI reference sequences: KDM2A, NP_001001984.2; KDM2A SF (*Homo sapiens*), NP_001243334.1; KDM2B, NP_001003953.1; KDM2B SF, NP_038938.1; FBXL19, NP_766336.2; CFP1, NP_083144.1; DNMT1, NP_001186360.2; MLL1, NP_001074518.1; MLL2, NP_083550.2; TET1, NP_001240786.1; TET3, NP_898961.2; IDAX, NP_001004367.2. GenBank®: MBD1, AAC68869.1; CXXC5, AAH89314.1. TET3 sequence from [122].

With the knowledge that methylated CpG dinucleotides are recognized by MBD proteins, it was proposed that non-methylated CpG dinucleotides may also act as a protein-binding site. To explore this possibility, Skalnik and colleagues conducted a phage-based ligand screen to discover protein factors that have the capacity to bind non-methylated CpGs [12]. From this screen, they identified a non-methylated CGBP (CpG-binding protein) whose DNA-binding activity relied on a cysteine-rich ZF-CxxC (zinc finger-CxxC) domain [12]. The discovery of CGBP and the demonstration that the ZF-CxxC domain is responsible for non-methylated CpG-binding activity motivated bioinformatic analyses that led to the identification of an extended family of ZF-CxxC domain-containing proteins (Figure 1 and Table 1). To reflect its discovery as the first ZF-CxxC-domain containing protein, CGBP was later renamed CFP1 (CxxC finger protein 1).

**Table 1:** ZF-CxxC domain-containing protein nomenclature

Gene name	CxxC nomenclature	Other names	NCBI Gene ID
*Kdm2a*	Cxxc8	Fbxl11, Jhdm1a, Ndy2	225876
*Kdm2b*	Cxxc2	Fbxl10, Jhdm1b, Ndy1	30841
*Fbxl19*	–	–	233902
*Cfp1*	Cxxc1	Cgbp, Phf18	74322
*Dnmt1*	Cxxc9	Met1	13433
*Mll1*	Cxxc7	All1, Htrx1, Kmt2a	214162
*Mll2*	–	Wbp7, Kmt2b	75410
*Mbd1*	Cxxc3	Pcm1	17190
*Tet1*	Cxxc6	Lcx	52463
*Tet3*	Cxxc10	–	194388
*Idax*	Cxxc4	–	319478
*Cxxc5*	Cxxc5	–	67393

**Figure 2:**
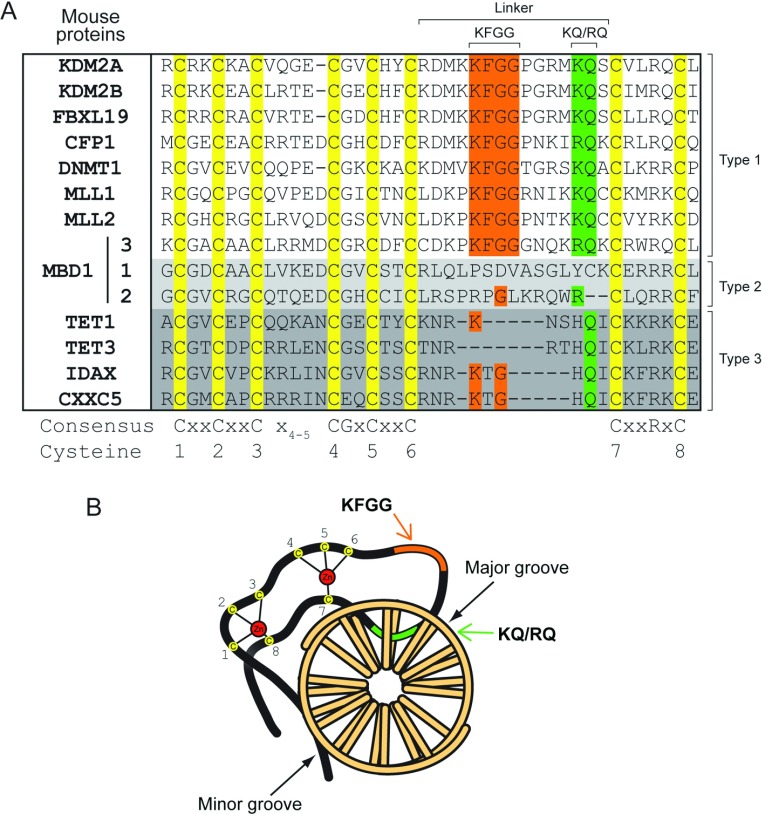
Primary sequence variation in ZF-CxxC domains (**A**) A manually curated multiple sequence alignment of all ZF-CxxC domains from mouse. ZF-CxxC domains can be split into three types depending on their sequence similarity, labelled as type-1, -2 and -3 on the right of the alignment. Eight cysteine residues are fully conserved across all of the ZF-CxxC domains (yellow). In the linker region between the two cysteine-rich clusters (1–6 and 7–8), a KFGG motif is conserved across the type-1 ZF-CxxC domains (orange) and a KQ or RQ DNA-binding motif which binds specifically to the CpG dinucleotide is present in all of the type-1 ZF-CxxC domains (green), but is lost in the type-2 ZF-CxxC domains and is HQ in the type-3 ZF-CxxC domains. Notably, whereas the type-3 ZF-CxxC domains are truncated in the linker region between the cysteine-rich clusters, the linker length is retained in the type-2 ZF-CxxC domains, but the sequence similarity to type-1 ZF-CxxC domains is completely lost. (**B**) A schematic of the ZF-CxxC domain highlighting the crescent structure and interaction with both the major and minor groove of DNA. Zn^2+^ ions (red) co-ordinate the eight cysteine residues (yellow). The KQ or RQ motif region (green) of the domain wedges into the major groove of the DNA, whereas the N-terminal (NT) and C-terminal (CT) regions of the ZF-CxxC domain interrogate the minor groove. The KFGG region (orange) forms part of the linker between the cysteine-rich regions, but does not from specific interactions with the CpG dinucleotide. DNA is viewed down the double helix with bases shown as rods.

The ZF-CxxC domain is characterized by two conserved cysteine-rich clusters which co-ordinate two Zn^2+^ ions intervened by a seemingly divergent sequence that effectively segregates the ZF-CxxC proteins into three distinct subtypes (Figure 2A). For the purposes of the present review, the three ZF-CxxC subtypes are referred to as type-1, -2 and -3. Proteins that encode type-1 ZF-CxxC domains include CFP1 and the histone H3 lysine 36 demethylases KDM2A and KDM2B. A recent series of studies have demonstrated that these proteins nucleate at CGIs *in vivo*, supporting the initial hypothesis that the ZF-CxxC domain may act as a CGI-targeting module [12–15]. However, the capacity of the ZF-CxxC domain to recognize CGIs in other family members, especially those in the type-2 and -3 subgroups, is less clear. In the present review, we examine our current understanding of ZF-CxxC domain structure followed by a more detailed discussion of the potential role that individual ZF-CxxC family members may play in CGI function.

## Structure of the ZF-CxxC domain

The short (35–42 amino acids) primary sequence of the ZF-CxxC domain and its conspicuous arrangement of ion-co-ordinating cysteine residues suggested, even without atomic resolution information, that the ZF-CxxC domain would form a compact DNA-binding module (Figure 2A). It was, however, by no means clear how this simple domain might provide such precise recognition of CpG dinucleotides and discriminate unmodified cytosine bases from the modified form which only differs by the presence of a single relatively inert methyl group. A recent succession of ZF-CxxC domain structures, in both unbound or DNA-associated states, has been instrumental in providing a detailed molecular and structural understanding of how this fascinating domain recognizes and interfaces with DNA [16–20]. These structures have also provided an important insight into why the type-1 and -3 ZF-CxxC domains possess unique DNA sequence-recognition properties.

Despite the sequence variation between type-1 and -3 ZF-CxxC domains, their overall domain architecture is highly similar. This is largely due to complete conservation of the two cysteine-rich clusters composed of CxxCxxCx_4/5_CGxCxxC and CxxRxC motifs (Figure 2A). The eight cysteine residues within these clusters co-ordinate two Zn^2+^ ions in a tetrahedral manner, stabilizing the ZF-CxxC domain in an extended crescent-shaped structure (Figure 2B). When bound to DNA, the ZF-CxxC domain lies perpendicular to the DNA axis and interrogates the major groove via a DNA-binding loop. Regions flanking the ZF-CxxC domain reach around to the opposite DNA face and interact with the minor groove (Figure 2B). By virtue of the fact that the ZF-CxxC domain essentially clamps around the DNA, it requires access to both the major and minor groove. This structural insight led to the realization that the ZF-CxxC domain must bind to linker regions of DNA between nucleosomes *in vivo*, as the physical association of DNA with histone octamers often prevents simultaneous access to the major and minor groove [21]. Therefore ZF-CxxC domain-mediated recognition of CGI DNA *in vivo* requires both the presence of non-methylated CpG dinucleotides and accessible internucleosomal DNA.

## Structural insights into DNA-binding specificity and capacity to discriminate between methylation states

**Figure 3:**
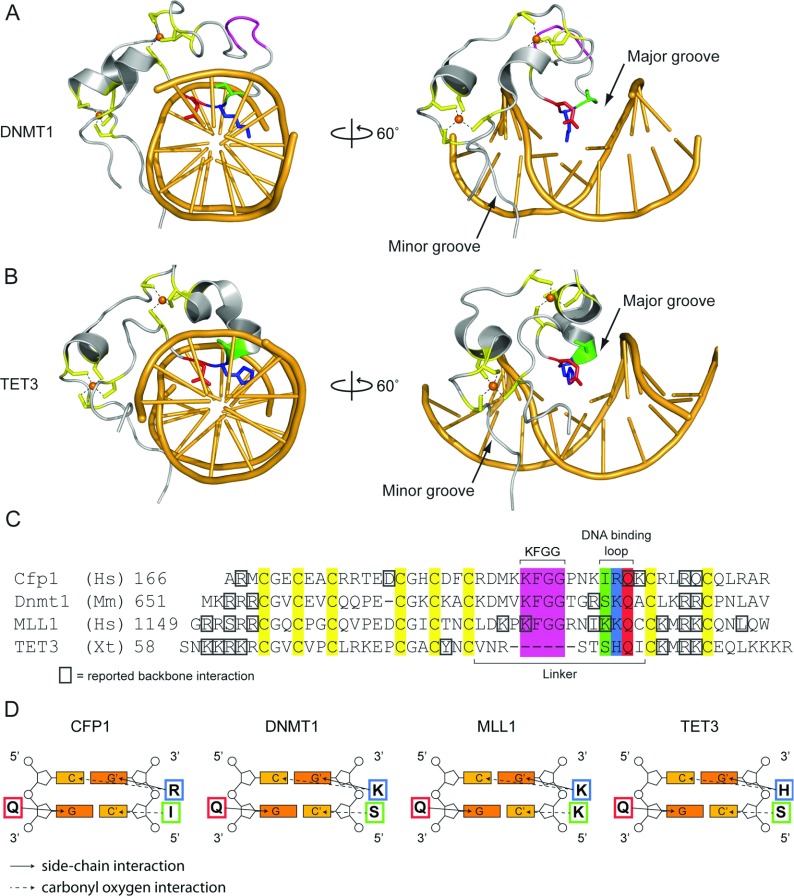
Structural insight into the DNA-binding properties of ZF-CxxC proteins (**A** and **B**) Crystal structures of the (**A**) *Homo sapiens* DNMT1 (PDB code 3PTA) and (**B**) *Xenopus tropicalis* TET3 (PDB code 4HP3) ZF-CxxC domains in complex with DNA, viewed down the double-helix axis (left) and rotated 60° to the right (right). For both structures, the eight cysteine residues are highlighted in yellow and their interaction with the Zn^2+^ ions (represented as orange spheres) are shown by dashes. The KFGG motif is highlighted in pink and the DNA-binding tripeptide is highlighted in green, blue and red for serine, lysine/histidine and glutamine respectively. The right-hand panels highlight that the DNA-binding tripeptide loop interrogates the CpG dinucleotide via the major groove of DNA, and that the N- and C-terminal parts of the ZF-CxxC domain interact with the minor groove. Zn^2+^ ions are represented as spheres. (**C**) A manually curated multiple sequence alignment of the amino acid sequence of four ZF-CxxC structures (PDB codes: CFP1, 3QMG; DNMT1, 3PTA; MLL, 2KKF; TET3, 4HP3). Residues reported to interact with the DNA backbone are marked with a grey box. Other residues are highlighted as in (**A**) and (**B**). (**D**) Schematic representations of the base-specific hydrogen bond interactions between the DNA-binding tripeptide and a CpG dinucleotide. Side-chain interactions are shown by a continuous arrow and carbonyl oxygen interactions are shown by a broken arrow.

**Figure 4:**
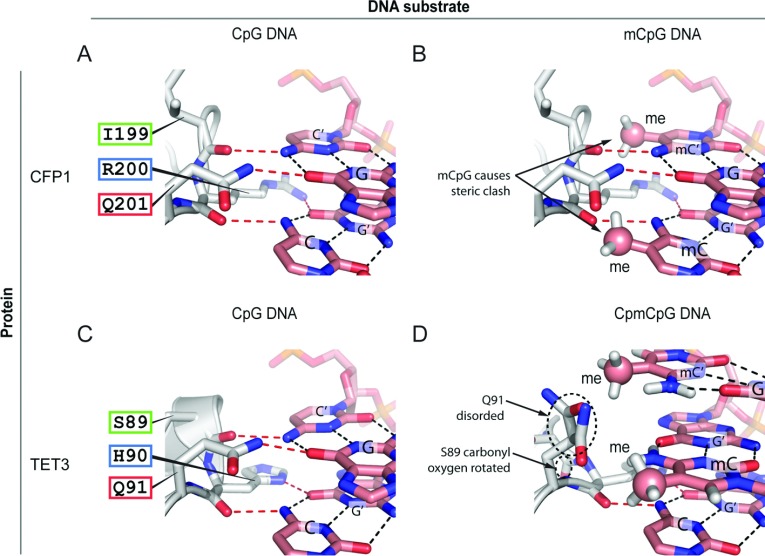
The effect of CpG methylation on DNA binding of type-1 and -3 ZF-CxxC domains (**A**) The CFP1 DNA-binding tripeptide IRQ (Ile-Arg-Gln) forms both side-chain and backbone hydrogen bonds with the CpG dinucleotide (CpG from one DNA strand and C'pG' from the other). Base-pairing hydrogen bonds are shown by black broken lines and ZF-CxxC tripeptide–DNA hydrogen bonds are shown as red broken lines. (**B**) As in (**A**), with methyl groups (me) at the 5 position of the cytosine rings. Cytosine methylation causes steric clash with the CFP1 tripeptide. (**C**) The TET1 DNA-binding tripeptide SHQ (Ser-His-Gln) forms both side-chain and backbone hydrogen bonds with the CpG dinucleotide as in (**A**). (**D**) DNA methylation of a CpCpG-containing substrate causes TET1 to shift binding 1 bp along the DNA to interact with the non-methylated cytosine.

Despite the overall structural similarities within the ZF-CxxC domain fold, type-1 and type-3 ZF-CxxC domains exhibit divergence at the DNA-binding interface, which appears to define their DNA-binding specificity (Figure 2A). In the type-1 ZF-CxxC domains, an extended linker region located between the two cysteine-rich motifs contains a highly conserved KFGG (Lys-Phe-Gly-Gly) motif. The available structures for CFP1, MLL (mixed lineage leukaemia protein) 1, KDM2A and DNMT1 (DNA methyltransferase 1) suggest that the KFGG motif is not involved in sequence-specific DNA interactions, but may be required to provide rigidity to the ZF-CxxC domain fold (Figures 3A and 3C). This KFGG motif is followed by a hydrophilic positively charged DNA-binding loop which penetrates the DNA major groove in a wedge-like manner [17,18] (Figures 3A and 3C). The ZF-CxxC domain makes a number of base-specific and phosphodiester backbone (Figure 3C) interactions with the DNA substrate. Most significantly, the conserved KQ (Lys-Gln) motif [RQ (Arg-Gln) in the case of CFP1] from type-1 domains makes specific side-chain and backbone interactions with the double-stranded CpG dinucleotide-recognition sequence, forming hydrogen bonds with the cytosine bases from both DNA strands and a guanine from one of the two strands (Figures 3C and 3D). The remaining guanine in the double-stranded CpG is interrogated by the amino acid immediately N-terminal to the KQ or RQ motif via the carbonyl oxygen of the peptide backbone (Figures 3C and 3D). In type-1 ZF-CxxC domains, DNA binding is therefore mediated by a rigid tripeptide-recognition module (Figure 4A). Importantly, the close proximity of the DNA-binding loop to the CpG dinucleotide substrate is such that cytosine methylation would create a severe steric clash at the DNA-binding interface (Figure 4B). Tight packing of adjacent helices and the nearby Zn^2+^ ion means that the DNA-binding tripeptide cannot undergo conformational change to accommodate the methyl moiety [18]. Consequently, in the presence of cytosine methylation, essential hydrogen bonds cannot form and DNA binding by the ZF-CxxC domain is prevented [17–19] (Figures 4A and 4B).

Interestingly, a recent structural study of the *Xenopus* TET (ten-eleven translocation) 3 type-3 ZF-CxxC domain revealed a more flexible mode of DNA binding that permits recognition of non-methylated cytosine bases in either a CpG or a non-CpG context. Similar to the type-1 domains described above, the type-3 ZF-CxxC domain of TET3 forms a crescent-like structure with a positively charged DNA-binding surface that wedges into the DNA major groove [20] (Figure 3B). However, the TET3 ZF-CxxC domain has a shortened linker before the DNA-binding loop that lacks the KFGG motif, whereas the DNA-binding interface contains an HQ (His-Gln) dipeptide corresponding to the KQ or RQ position of the type-1 domains (Figure 3C). Despite these differences, the TET3 ZF-CxxC domain bound a non-methylated CpG dinucleotide in an ACGT context (Figures 3B, 3D and 4C). A second structure of the TET3 ZF-CxxC domain bound to a DNA molecule containing a non-methylated cytosine followed by a methylated CpG dinucleotide (Cm**C**GG) revealed a unique capacity for the type-3 ZF-CxxC domain to interact with unmodified cytosine in a non-CpG context. In this sequence, the ZF-CxxC domain shifts one nucleotide along to interact with the non-methylated cytosine (Figure 4D). This shift leads to a steric clash between the methyl group and the Gln^91^ side chain from the HQ motif, causing the Gln^91^ and Ser^89^ residues to become partially disordered and lose hydrogen-bonding with the DNA [20] (Figure 4D). Importantly, owing to the shortened linker region preceding the DNA-binding loop and loss of stabilizing hydrogen bonds (for example between Asp^189^ and the DNA-binding loop in CFP1), the DNA-binding interface of the TET3 ZF-CxxC domain is not as rigid as those found in type-1 ZF-CxxC domains. The increased flexibility that this confers allows TET3 to seemingly recognize non-methylated cytosine bases in a broader range of sequence contexts, albeit with a slight preference for CpG [20].

## From binding non-methylated DNA *in vitro* to CpG island recognition and chromatin modification *in vivo*

*In vitro* binding analyses and structural studies have provided a molecular description of how the ZF-CxxC domain recognizes its DNA substrates. In most cases, these studies predict that ZF-CxxC domains should associate with non-methylated CGIs *in vivo*. Nevertheless, it has taken more than a decade since the discovery of CFP1 (CGBP) to convincingly demonstrate at the genome-scale that the ZF-CxxC domain can function as a CGI-targeting module [13,14]. In the following sections, we consider each of the individual ZF-CxxC domain-containing proteins and outline our current understanding of their DNA-binding properties and function *in vivo*.

### KDM2A, KDM2B and FBXL19 (F-box and leucine-rich repeat protein 19)

**Figure 5:**
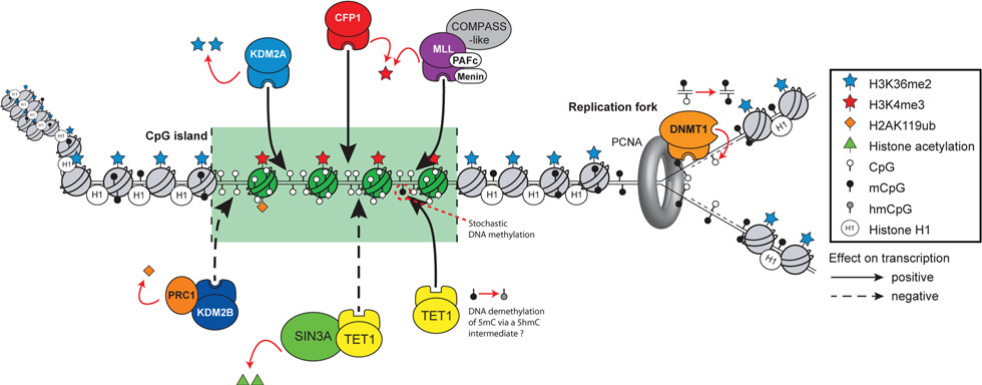
The role of ZF-CxxC proteins at CGIs and during DNA replication A schematic representation of the convergence of ZF-CxxC domain-containing protein function at CGI elements and the role of the ZF-CxxC domain-containing DNMT1 during DNA replication. PAFC, RNA polymerase-associated factor complex; PCNA, proliferating-cell nuclear antigen.

KDM2A is a JmjC (Jumonji C) domain-containing histone lysine demethylase enzyme which catalyses removal of methylation from histone H3 Lys^36^ with a preference for the dimethyl modification state (H3K36me2) [22]. In addition to the JmjC domain, KDM2A also encodes a type-1 ZF-CxxC domain that binds specifically to DNA containing non-methylated CpGs *in vitro* [13]. KDM2A is significantly enriched at more than 90% of CGIs genome-wide in mouse ESCs (embryonic stem cells) [13] (Figure 5). Importantly, this includes CGI promoters of both expressed and non-expressed genes, suggesting that its nucleation on chromatin is dependent on recognition of non-methylated DNA as opposed to the transcriptional state of the associated gene.

H3K36me2, the substrate for KDM2A, is one of the most abundant histone modifications in mammalian cells, being found on 30–50% of total histone H3 and localizing to both inter- and intra-genic regions [23–25]. Importantly, KDM2A-bound CGIs are depleted of H3K36me2 and RNAi (RNA interference)-mediated knockdown of KDM2A results in increased H3K36me2 at these regions, suggesting that KDM2A plays an active role in removing H3K36me2 from CGIs [13]. Although the function of H3K36me2 remains poorly understood, in *Saccharomyces cerevisiae* H3K36me2 appears to be inhibitory to transcriptional initiation. This is in part thought to be mediated through binding of the EAF3 chromodomain-containing protein to H3K36me2 and recruitment of the HDAC-containing RPD3S co-repressor complex [26,27]. Furthermore, it was demonstrated recently that H3K36 methylation can inhibit the interaction between histone chaperones and histone H3, effectively blocking histone exchange on chromatin and perhaps supressing further the capacity for non-regulatory regions to support transcriptional initiation [28]. Although it has yet to be unequivocally demonstrated that H3K36me2 leads to similar transcriptional repression in higher eukaryotes, pervasive H3K36me2 in the mammalian genome suggests that this modification may also contribute to the suppression of erroneous transcription initiation. Therefore it is tempting to speculate that targeting of KDM2A to CGIs, via its ZF-CxxC domain, leads to a specific depletion of H3K36me2 at CGIs, which could in turn help to create a favourable chromatin environment for initiation of transcription.

KDM2B, a paralogue of KDM2A, possesses an almost identical domain architecture including a type-1 ZF-CxxC domain (Figure 1). Similarly to KDM2A, KDM2B removes H3K36me2 [29] and can contribute to cellular immortalization, transformative capacity in cancer and reprogramming [29–33]. Recent ChIP-seq (chromatin immunoprecipitation sequencing)-based analysis indicates that KDM2B binds to CGIs genome-wide in a manner similar to that of KDM2A [13,15,34] (Figure 5). Intriguingly, detailed inspection of KDM2A- and KDM2B-binding profiles revealed a unique subset of CGIs that were preferentially enriched for KDM2B and depleted of KDM2A. These CGIs were generally associated with genes involved in embryo development, morphogenesis and cellular differentiation. In mouse ESCs, these type of genes are often bound by the PRCs (polycomb group repressive complexes) that function as transcriptional repressors [35], suggesting that KDM2B may contribute to polycomb-mediated transcriptional repression [15].

In mammals, the highly conserved polycomb system consists of two central PRCs called PRC1 and PRC2 [35,36]. Interestingly, PRCs appear to function almost exclusively at CGI elements, yet the mechanisms governing their recruitment to these sites remains poorly defined. The absence of an apparent sequence-specific DNA-binding domain within components of the canonical PRC1 and PRC2 complexes has led to the proposal that transient transcription factor or non-coding RNA-based interactions may provide a mechanism for targeting to CGIs [35]. Interestingly, experiments in cancer cells indicated that KDM2B associates with a variant PRC1 complex containing BCoR (Bcl-6-interacting co-repressor), PCGF1 [polycomb group RING (really interesting new gene) finger 1], RYBP [RING and YY1 (Yin and Yang 1)-binding protein], YAF2 (YY1-associated factor 2) and RING1B [37–39]. A similar complex was also purified from non-transformed mouse ESCs, suggesting that this variant PRC1 complex has a biological role in a non-malignant context [15]. On the basis of the ZF-CxxC-dependent capacity of KDM2B to recognize non-methylated CGI DNA and its enrichment at polycomb-occupied CGIs, it was hypothesized that KDM2B may contribute to recruitment of PRC1 to these sites (Figure 5). Indeed, knockdown of KDM2B using an shRNA (short hairpin RNA)-based approach caused a reduction in the levels of RING1B at polycomb target sites genome-wide, with a concomitant increase in expression of some polycomb-repressed genes [15,34,39a].

Although polycomb-repressed genes account for a relatively small subset of CGIs [36], KDM2B is present at virtually all CGIs through its ZF-CxxC-dependent recognition of non-methylated DNA. Interestingly, genome-resolution RING1B ChIP-seq analysis revealed that, in addition to the previously characterized CGIs known to be occupied by high levels of PRC1, the majority of other CGIs in the genome also show low magnitude, yet appreciable enrichment of PRC1 [15]. Binding of PRC1 to these low-magnitude sites is dependent on KDM2B, suggesting that this targeting relies on recognition of non-methylated DNA. Therefore it appears that KDM2B recruits PRC1 at low levels to CGIs genome-wide, possibly as a sampling mechanism for gene repression. It seems reasonable to hypothesize that, when this sampling module encounters the appropriate chromatin environment, possibly created by a lack of activating transcription factors, accumulation of PRCs can occur and transcriptional repression can be achieved.

The type-1 ZF-CxxC domain-containing protein FBXL19 is highly similar to KDM2A and KDM2B, with the exception that it lacks the N-terminal JmjC domain (Figure 1). Interestingly, both the *KDM2A* and *KDM2B* genes also have alternative transcription start sites downstream of their JmjC domain, giving rise to short forms of these proteins that closely resemble FBXL19 (Figure 1). The role of FBXL19 and the short forms of KDM2A and KDM2B remain poorly defined, but the presence of a presumably functional ZF-CxxC domain in each suggests that they probably recognize and affect CGI function.

### CFP1

CFP1 encodes a type-1 ZF-CxxC domain and is essential for early mouse development [40]. The failure of CFP1-null ESCs to effectively differentiate *in vitro* is consistent with an important role for CFP1 in lineage commitment and perhaps relates to its capacity to bind CGIs and contribute to gene regulation [41]. The CFP1 protein is a component of the mammalian SETD1 (SET domain 1) H3K4 methyltransferase complex, which includes SETD1A or SETD1B, ASH2L (absent, small or homeotic 2-like), RbBP5 (retinoblastoma-binding protein 5), WDR (WD40 repeat) 5 and WDR82, and DPY-30 (dosage compensation protein 30) [42–44]. The SETD1 complex places H3K4 di- and tri-methylation (H3K4me2 and me3) [42,43]. These histone modifications are generally associated with the 5′ ends of genes [36,45–47], consistent with the localization of CFP1 [14,48]. Although the precise molecular function of H3K4me2/3 *in vivo* and its contribution to gene expression remain poorly defined, these marks are generally considered permissive to active transcription. This may be achieved by the recruitment of specific PHD (plant homeodomain) or tudor domain effector proteins [49–53].

Genome-wide binding studies in mouse brain tissue demonstrated that CFP1 associates with more than 80% of CGIs, and almost all CFP1-bound CGIs exhibit significant enrichment of H3K4me3 [14] (Figure 5). Similarly to KDM2A, localization of CFP1 to CGIs did not depend on the transcriptional state of the associated gene, suggesting that ZF-CxxC domain-mediated recognition of non-methylated DNA was primarily responsible for the chromatin-binding profiles of CFP1. Consistent with this observation, an exogenous CpG-rich DNA sequence lacking gene-regulatory features can recruit CFP1 and nucleate H3K4me3, apparently in the absence of transcription factors and RNAPII (RNA polymerase II) [14]. Interestingly, a subset of non-methylated CGIs associated with polycomb-mediated repression were not enriched for CFP1 [14], suggesting that, in some instances, the chromatin architecture at specific CGIs may restrict access of the ZF-CxxC domain.

In keeping with a role for CFP1 in targeting H3K4 methylation, mouse ESCs with constitutively deleted CFP1 exhibit a loss of H3K4me3 at up to half of CGIs in the mouse genome [54]. Somewhat surprisingly, however, loss of H3K4me3 was most prevalent at highly transcribed promoters and CFP1-null ESCs reconstituted with a mutant version of CFP1 lacking a functional ZF-CxxC domain restored normal H3K4me3 levels at affected genes [54]. This suggests that, in mouse ESCs, CFP1 can guide H3K4me3 to appropriate target sites in a manner that is independent of its DNA-binding activity, possibly through the activity of the CFP1 PHD domain which was shown recently to bind H3K4 methylation [55]. If the PHD domain is responsible for ZF-CxxC domain-independent targeting of CFP1 to CGIs, this would presumably require appropriate H3K4 methylation to be initiated at CGIs through alternative mechanisms. An intriguing possibility is that other H3K4 methyltransferases such as MLL1 or MLL2, which also encode ZF-CxxC domains, may fulfil this requirement.

In addition to H3K4me3 loss at CGI promoters, CFP1-null ESCs also appear to mistarget H3K4me3 methylation. The resulting ‘ectopic’ H3K4me3 peaks appear at numerous intergenic regions of the genome, and genes within the vicinity of these new H3K4me3 sites often displayed increased transcription [54]. Reintroduction of wild-type CFP1 abolished these ectopic H3K4me3 peaks, whereas a ZF-CxxC mutant did not. Therefore it appears that ZF-CxxC-independent mechanisms are capable of recruiting CFP1 to highly transcribed CGIs, whereas the ZF-CxxC domain of CFP1 is necessary for retention of the SETD1 complex at CGIs and to prevent its mis-localization to other regions of the genome.

### MLL1 and MLL2

In addition to CFP1-containing SETD1 complexes, links between the mammalian H3K4 methylation systems and recognition of non-methylated DNA via ZF-CxxC domains extends to the MLL family. The MLL H3K4 methyltransferase family comprises four large proteins (MLL1–MLL4) that form independent multisubunit complexes that share a set of interaction partners with the SETD1 complexes, including ASH2L, WDR5, RbBP5 and DPY-30 [56]. MLL1 (also known as ALL-1, HRX, CXXC7 or KMT2A) and MLL2 (also known as MLL4, WBP7 or KMT2B) are closely related proteins that appear to have arisen through an evolutionary gene-duplication event [57,58]. They both encode a type-1 ZF-CxxC domain (Figure 1), whereas MLL3 and MLL4 lack ZF-CxxC domains. The ZF-CxxC domains of MLL1 and MLL2 bind non-methylated DNA *in vitro* [59,60], but how they contribute to localization *in vivo* is not fully understood.

MLL1 plays an essential role in early mammalian development and in definitive haemopoiesis [61,62]. At a molecular level, MLL1 localizes to approximately 5000 gene promoters in human lymphoma cells, highly coincident with H3K4me3, RNAPII and active transcription [63]. MLL1 is also enriched across the *HoxA* cluster, a GC-rich genomic region exhibiting numerous CGIs [63,64] (Figure 5). Therefore MLL1 localization exhibits hallmarks of ZF-CxxC-mediated recruitment, but, unlike KDM2 and CFP1 proteins [13–15], is restricted to a subset of CGI elements that are actively transcribed (Figure 5). Similarly menin, an N-terminal binding partner of MLL1 [65], associates with the 5′ end of approximately 2000 genes in a variety of cell types, frequently coinciding with MLL1-binding sites, H3K4me3 modification and high levels of gene expression [66]. The restriction of MLL1, and its binding partner menin, to a subset of CGIs suggests that mechanisms independent of ZF-CxxC-mediated non-methylated targeting may play a role in MLL1 localization [64]. This more restricted binding pattern could be due to the activity of other chromatin-binding modules, including the N-terminal AT hooks of MLL1 which have been demonstrated to bind AT-rich regions of DNA [67] and a PHD finger (PHD3) which may recognize specific histone methylation marks [68–71]. Similarity, non-histone protein–protein interactions may also influence MLL1 localization. For example, members of PAF1C (RNA polymerase-associated factor 1 complex) interact with the CXXC domain of MLL1 [70,72]. Together, this complement of chromatin-binding activities probably shapes how MLL1 is recruited to appropriate target sites *in vivo*.

Chromosomal translocations that couple MLL1 to one of more than 60 known fusion partners have been implicated in driving aggressive adult and childhood leukaemias [73]. These translocation events result in the N-terminal portion of the *MLL1* gene, including the AT-hooks and ZF-CxxC domain, being fused to the C-terminal portion of a translocation partner [65,74,75]. One of the most common MLL1 translocation events creates an MLL–AF9 (ALL1–fused gene from chromosome 9 protein) fusion protein [76]. AF9 is a component of SEC (super-elongation complex) [77,78], which contributes to transcriptional elongation. The MLL–AF9 fusion protein appears to result in aberrant targeting of SEC to normally silent MLL target genes, causing deleterious expression of these genes. Other MLL fusion proteins also affect target gene expression, but are thought to achieve this by recruitment of histone-modifying activities [77,79]. Interestingly, MLL–AF9 with a mutant ZF-CxxC domain exhibited severely reduced transforming potential [17,70,74], suggesting that the ZF-CxxC domain plays a crucial role in directing leukaemogenic fusion proteins to genomic targets.

MLL2 plays an essential role in early development, with MLL2 deletion causing embryonic lethality in mice at E10.5 (embryonic day 10.5) [80]. Despite having almost identical domain architecture and forming similar H3K4 methyltransferase complexes, MLL1 and MLL2 display some non-redundant functions [81,82]. For example, MLL2 is required for gametogenesis and also briefly in the zygote as a maternally derived factor [82,83]. Furthermore, MLL2 loss in macrophages causes gene-specific loss of H3K4me3 and loss of LPS (lipopolysaccharide)-triggered intracellular signalling [81]. Intriguingly, MLL2-fusion proteins have not been implicated in leukaemogenesis, which is perhaps surprising given that MLL1 and MLL2 have highly conserved ZF-CxxC domains and seemingly identical DNA-binding activities *in vitro* [16,60] (Figure 2A). This is exemplified by the observation that a synthetic MLL2–ENL (eleven-nineteen leukaemia) fusion protein was unable to transform haemopoietic cells, whereas a similar MLL1–ENL fusion is leukaemogenic [60]. Domain-swap experiments producing various MLL1 or MLL2 hybrid ENL fusions suggest that the ZF-CxxC domain and immediate flanking regions may be subtly different between MLL1 and MLL2, such that MLL2 fusions lack transforming potential [60].

### DNMT1

DNMT1 is a large modular protein composed of a RFTS (replication foci-targeting sequence), a type-1 ZF-CxxC domain, a pair of BAH (bromo-adjacent homology) domains (BAH1 and BAH2), and a C-terminal catalytic domain (Figure 1). DNMT1 associates with PCNA (proliferating-cell nuclear antigen) at replication forks via its RFTS [84] where it copies pre-existing parental methylation patterns on to newly replicated daughter strands of DNA. During DNA replication, symmetrically methylated CpG dinucleotides become hemimethylated as a result of semiconservative replication. Following replication, DNMT1 must recognize these sites and faithfully reinstate symmetrical methylation [85] (Figure 5). To achieve this, DNMT1 catalyses addition of a methyl group to hemimethylated CpG dinucleotides with an efficiency 30–50-fold greater than for unmodified CpGs [84,86]. In part, its substrate specificity *in vivo* is dictated by a protein partner called UHRF1 (ubiquitin-like with PHD and RING finger domains 1) that recognizes hemimethylated CpGs and is essential for correct targeting of DNMT1 [87–90].

The presence of a functional type-1 ZF-CxxC domain in DNMT1 [91] is perhaps somewhat surprising and counterintuitive given that the vast majority of CGIs are free of DNA methylation and the main substrate for DNMT1 is hemimethylated DNA. Nevertheless, a recent structural study provided a potentially interesting suggestion for how the ZF-CxxC domain of DNMT1 might function to limit DNMT1 to appropriate substrates [19]. By solving the crystal structure of a truncated form of DNMT1 in complex with DNA containing non-methylated CpGs [19], it became apparent that, when DNMT1 is bound to non-methylated CpG DNA, the ZF-CxxC domain occludes access of the DNMT1 catalytic site to the CpG dinucleotide. Furthermore, a highly acidic polypeptide loop which connects the ZF-CxxC domain to the BAH1 domain (termed the autoinhibitory linker) blocks the DNMT1 active-site cleft [19]. This led to the suggestion that, when DNMT1 encounters an appropriate DNA substrate containing hemimethylated CpGs, the ZF-CxxC domain is unable to bind, causing the autoinhibitory loop to adopt an alternative conformation that renders the active site accessible. In support of this model, deletion of the ZF-CxxC domain and autoinhibitory linker increases the catalytic activity of DNMT1 specifically on non-methylated, but not hemimethylated, DNA substrates [19].

The ZF-CxxC-dependent autoinhibitory model was based on the study of a truncated form of DNMT1 that does not include the N-terminal RFTS domain. A subsequent analysis of full-length DNMT1 revealed that the ZF-CxxC domain did not influence its preference for hemimethylated over unmodified DNA substrates [92]. This observation was supported by structural studies using larger DNMT1 fragments that suggest that the RFTS domain can insert into the DNMT1 DNA-binding pocket and play an inhibitory role that prevails over the autoinhibitory linker implicated from the previous structural studies using smaller DNMT1 fragments [93,94]. Together, these studies suggest that DNMT1 has several in-built properties that help to limit its catalytic activity, with contributions from both ZF-CxxC domain-dependent and -independent mechanisms.

### MBD1

The transcriptional repressor MBD1 encodes an MBD capable of recognizing methylated CpGs [95–97] and three ZF-CxxC domains (Figure 1). Two of the MBD1 ZF-CxxC domains (CxxC-1 and CxxC-2) are type-2 domains which lack a functional DNA-binding loop [95] (Figure 2A) and instead appear to function as protein–protein interaction modules [98,99]. The third ZF-CxxC domain (CxxC-3) is a type-1 domain capable of binding to non-methylated CpG dinucleotides *in vitro* [95,96]. The combination of both ZF-CxxC and MBDs in MBD1 suggests that it could potentially read non-methylated and methylated CpG dinucleotides individually or in combination [95]. However, point mutations in the CxxC-3 domain which disrupt DNA binding *in vitro* did not affect the recruitment of MBD1, suggesting that functional MBD1 targeting can be achieved in the absence of the ZF-CxxC domain [96]. Interestingly, in DNMT-null cells, where DNA methylation is lost, the DNA-binding capacity of the CxxC-3 domain results in targeting of MBD1 to non-methylated heterochromatic foci. It is therefore possible that the MBD1 CxxC-3 domain may act as a relevant targeting module in specific instances where DNA methylation levels are drastically reduced, for example, in pre-implantation embryos [96]. Nevertheless, in the majority of cases where the genome is pervasively methylated, the MBD appears to play a dominant role in guiding MBD1 to methylated DNA [96].

### TET1 and TET3

Recently, it has become apparent that vertebrate genomes contain small yet significant levels of 5hmC (5-hydroxymethylcytosine) [100–103]. 5hmC is generated by oxidation of 5mC (5-methylcytosine) by the TET1–TET3 protein family [100,101] in an Fe(II)- and 2-oxoglutarate- (α-ketoglutarate) dependent manner. The capacity of TET proteins to convert 5mC into 5hmC prompted speculation that the TET proteins may form part of a mammalian DNA demethylation system [101,104]. In addition to the catalytic DSBH (double-stranded β-helix) domain, TET1 and TET3 encode an N-terminal ZF-CxxC domain (Figure 1). TET2 lacks a ZF-CxxC domain; however, the neighbouring IDAX (inhibition of the Dvl and axin complex protein) (CXXC4) protein has a ZF-CxxC domain which is very similar to those in TET1 and TET3 (Figure 2A), suggesting that TET2 and IDAX may have arisen from a duplication and partial inversion of either TET1 or TET3.

The three TET enzymes have distinct expression patterns and exhibit different phenotypes upon genetic perturbation, suggesting that they may have unique functions during development and in specific cell types. TET1 is highly expressed in mouse ESCs [101] where it maintains the pluripotent state by regulating the expression of pluripotency factors [105–107]. TET1 has also been implicated in the establishment of pluripotency during iPS (induced pluripotent stem) cell reprogramming [108] and in the control of meiosis in female germ cells [109], again suggesting a role in cell fate decisions. In conflict with these reported roles for TET1 in pluripotency, other studies have failed to observe a loss of pluripotency upon knockdown of TET1, but did observe skewed differentiation [110,111]. Furthermore, TET1-null mouse ESCs remain undifferentiated and express pluripotency factors, but again display skewed differentiation [112]. These discrepancies may be explained by off-target effects of shRNAs [113], or that the phenotypes observed during acute TET1 loss are different from those seen during chronic loss of TET1 in the knockout mouse model [112]. Unlike TET1, TET3 expression is mostly restricted to the oocyte and zygote, where it appears to contribute to either rapid demethylation or conversion of 5mC into 5hmC in the male pronucleus after fertilization [104] and ultimately TET3 neonatal lethality [114]. The recent generation of TET1 and TET2 double-knockout mice revealed that they are viable and overtly normal. The lack of a severe phenotype in these mice may be due to compensatory affects contributed by TET3 during development [115].

The type-3 ZF-CxxC domains found in TET1 and TET3 differ from type-1 domains, as they exhibit a truncated linker region and a divergent DNA-binding loop (Figure 2A). The consequences of these differences are not fully understood, although a recent study suggests that TET3 can recognize non-methylated cytosine bases in any sequence context with a slight preference for CpG [20]. In contrast, it has been reported that the TET1 ZF-CxxC domain binds CpGs irrespective of methylation status [116,117] or that it lacks sequence-specific DNA-binding activity [118]. Despite these conflicting claims, *in vivo* evidence suggests that, at least in some instances, TET ZF-CxxC domains may constitute CGI-targeting modules. A number of independent studies have profiled TET1 localization genome-wide in mouse ESCs [111,117,119] and all generally concluded that TET1 is preferentially enriched at gene promoters, with moderate enrichment in the exons of genes. Importantly, TET1 enrichment shows a strong positive correlation with CpG density, consistent with a potential ZF-CxxC-dependent CGI-targeting mechanism (Figure 5). Furthermore, mutation of the TET1 ZF-CxxC domain prevented interaction with CGI DNA in an *in vitro* pull-down assay [117].

On the basis of the enzymatic activity of TET proteins, there has been intense focus on determining where 5hmC is found in the genome. Genome-wide mapping studies using a variety of approaches have suggested that 5hmC is enriched at gene promoters with intermediate to high CpG density [111,117,119], bivalent promoters [111,117,119–121], within gene bodies [117] and promoters [106] of actively expressed, genes and at *cis*-regulatory elements [106,120,121]. Somewhat counterintuitively, CpG-rich promoters which exhibit the highest levels of TET1 appear to be largely devoid of 5hmC. This may be because the function of TET protein nucleation at CGIs is to ‘mop up’ aberrantly placed 5mC by conversion into 5hmC and perhaps subsequent reversal to the non-methylated state (Figure 5). In support of this contention, knockdown of TET1 results in acquisition of DNA methylation at specific CGIs [117]. Alternatively, it has also been reported that, for some genomic targets, TET1 has a repressive role that is independent of 5hmC involving direct recruitment of the SIN3A co-repressor complex [111] (Figure 5). Clearly, further study is required to fully understand the role of the ZF-CxxC domain in TET protein enzymatic function particularly with respect to its proposed role in counteracting DNA methylation.

## Conclusions

In order to fully understand the contribution of CGIs to gene expression, an important future challenge is to elucidate the influence that ZF-CxxC proteins have on CGI function. Although there has been a significant amount of progress in this area over the last few years, clearly a more defined grasp of ZF-CxxC DNA-binding specificity and detailed understanding of ZF-CxxC domain-containing protein localization and function *in vivo* are essential in achieving this goal.

## References

[1] Bird A.P. (1980). DNA methylation and the frequency of CpG in animal DNA. Nucleic Acids Res..

[2] Deaton A.M., Bird A. (2011). CpG islands and the regulation of transcription. Genes Dev..

[3] Lewis J.D., Meehan R.R., Henzel W.J., Maurer-Fogy I., Jeppesen P., Klein F., Bird A. (1992). Purification, sequence, and cellular localization of a novel chromosomal protein that binds to methylated DNA. Cell.

[4] Meehan R.R., Lewis J.D., McKay S., Kleiner E.L., Bird A.P. (1989). Identification of a mammalian protein that binds specifically to DNA containing methylated CpGs. Cell.

[5] Klose R.J., Bird A.P. (2006). Genomic DNA methylation: the mark and its mediators. Trends Biochem. Sci..

[6] Bell A.C., Felsenfeld G. (2000). Methylation of a CTCF-dependent boundary controls imprinted expression of the *Igf2* gene. Nature.

[7] Jones P.A. (2012). Functions of DNA methylation: islands, start sites, gene bodies and beyond. Nat. Rev. Genet..

[8] Bird A., Taggart M., Frommer M., Miller O.J., Macleod D. (1985). A fraction of the mouse genome that is derived from islands of nonmethylated, CpG-rich DNA. Cell.

[9] Gardiner-Garden M., Frommer M. (1987). CpG islands in vertebrate genomes. J. Mol. Biol..

[10] Saxonov S., Berg P., Brutlag D.L. (2006). A genome-wide analysis of CpG dinucleotides in the human genome distinguishes two distinct classes of promoters. Proc. Natl. Acad. Sci. U.S.A..

[11] Long H.K., Sims D., Heger A., Blackledge N.P., Kutter C., Wright M.L., Grutzner F., Odom D.T., Patient R., Ponting C.P., Klose R.J. (2013). Epigenetic conservation at gene regulatory elements revealed by non-methylated DNA profiling in seven vertebrates. eLife.

[12] Voo K.S., Carlone D.L., Jacobsen B.M., Flodin A., Skalnik D.G. (2000). Cloning of a mammalian transcriptional activator that binds unmethylated CpG motifs and shares a CXXC domain with DNA methyltransferase, human trithorax, and methyl-CpG binding domain protein 1. Mol. Cell. Biol..

[13] Blackledge N.P., Zhou J.C., Tolstorukov M.Y., Farcas A.M., Park P.J., Klose R.J. (2010). CpG islands recruit a histone H3 lysine 36 demethylase. Mol. Cell.

[14] Thomson J.P., Skene P.J., Selfridge J., Clouaire T., Guy J., Webb S., Kerr A.R., Deaton A., Andrews R., James K.D. (2010). CpG islands influence chromatin structure via the CpG-binding protein Cfp1. Nature.

[15] Farcas A.M., Blackledge N.P., Sudbery I., Long H.K., McGouran J.F., Rose N.R., Lee S., Sims D., Cerase A., Sheahan T.W. (2012). KDM2B links the polycomb repressive complex 1 (PRC1) to recognition of CpG islands. eLife.

[16] Allen M.D., Grummitt C.G., Hilcenko C., Min S.Y., Tonkin L.M., Johnson C.M., Freund S.M., Bycroft M., Warren A.J. (2006). Solution structure of the nonmethyl-CpG-binding CXXC domain of the leukaemia-associated MLL histone methyltransferase. EMBO J..

[17] Cierpicki T., Risner L.E., Grembecka J., Lukasik S.M., Popovic R., Omonkowska M., Shultis D.D., Zeleznik-Le N.J., Bushweller J.H. (2010). Structure of the MLL CXXC domain–DNA complex and its functional role in MLL-AF9 leukemia. Nat. Struct. Mol. Biol..

[18] Xu C., Bian C., Lam R., Dong A., Min J. (2011). The structural basis for selective binding of non-methylated CpG islands by the CFP1 CXXC domain. Nat. Commun..

[19] Song J., Rechkoblit O., Bestor T.H., Patel D.J. (2011). Structure of DNMT1–DNA complex reveals a role for autoinhibition in maintenance DNA methylation. Science.

[20] Xu Y., Xu C., Kato A., Tempel W., Abreu J.G., Bian C., Hu Y., Hu D., Zhao B., Cerovina T. (2012). Tet3 CXXC domain and dioxygenase activity cooperatively regulate key genes for *Xenopus* eye and neural development. Cell.

[21] Zhou J.C., Blackledge N.P., Farcas A.M., Klose R.J. (2012). Recognition of CpG island chromatin by KDM2A requires direct and specific interaction with linker DNA. Mol. Cell. Biol..

[22] Tsukada Y., Fang J., Erdjument-Bromage H., Warren M.E., Borchers C.H., Tempst P., Zhang Y. (2006). Histone demethylation by a family of JmjC domain-containing proteins. Nature.

[23] Peters A.H., Kubicek S., Mechtler K., O’Sullivan R.J., Derijck A.A., Perez-Burgos L., Kohlmaier A., Opravil S., Tachibana M., Shinkai Y. (2003). Partitioning and plasticity of repressive histone methylation states in mammalian chromatin. Mol. Cell.

[24] Garcia B.A., Thomas C.E., Kelleher N.L., Mizzen C.A. (2008). Tissue-specific expression and post-translational modification of histone H3 variants. J. Proteome Res..

[25] Robin P., Fritsch L., Philipot O., Svinarchuk F., Ait-Si-Ali S. (2007). Post-translational modifications of histones H3 and H4 associated with the histone methyltransferases Suv39h1 and G9a. Genome Biol..

[26] Li B., Jackson J., Simon M.D., Fleharty B., Gogol M., Seidel C., Workman J.L., Shilatifard A. (2009). Histone H3 lysine 36 dimethylation (H3K36me2) is sufficient to recruit the Rpd3s histone deacetylase complex and to repress spurious transcription. J. Biol. Chem..

[27] Carrozza M.J., Li B., Florens L., Suganuma T., Swanson S.K., Lee K.K., Shia W.J., Anderson S., Yates J., Washburn M.P., Workman J.L. (2005). Histone H3 methylation by Set2 directs deacetylation of coding regions by Rpd3S to suppress spurious intragenic transcription. Cell.

[28] Venkatesh S., Smolle M., Li H., Gogol M.M., Saint M., Kumar S., Natarajan K., Workman J.L. (2012). Set2 methylation of histone H3 lysine 36 suppresses histone exchange on transcribed genes. Nature.

[29] He J., Kallin E.M., Tsukada Y., Zhang Y. (2008). The H3K36 demethylase Jhdm1b/Kdm2b regulates cell proliferation and senescence through p15(Ink4b). Nat. Struct. Mol. Biol..

[30] Pfau R., Tzatsos A., Kampranis S.C., Serebrennikova O.B., Bear S.E., Tsichlis P.N. (2008). Members of a family of JmjC domain-containing oncoproteins immortalize embryonic fibroblasts via a JmjC domain-dependent process. Proc. Natl. Acad. Sci. U.S.A..

[31] Liang G., He J., Zhang Y. (2012). Kdm2b promotes induced pluripotent stem cell generation by facilitating gene activation early in reprogramming. Nat. Cell Biol..

[32] Wang T., Chen K., Zeng X., Yang J., Wu Y., Shi X., Qin B., Zeng L., Esteban M.A., Pan G., Pei D. (2011). The histone demethylases Jhdm1a/1b enhance somatic cell reprogramming in a vitamin-C-dependent manner. Cell Stem Cell.

[33] He J., Nguyen A.T., Zhang Y. (2011). KDM2b/JHDM1b, an H3K36me2-specific demethylase, is required for initiation and maintenance of acute myeloid leukemia. Blood.

[34] Wu X., Johansen, Jens V., Helin K. (2013). Fbxl10/Kdm2b recruits polycomb repressive complex 1 to CpG islands and regulates H2A ubiquitylation. Mol. Cell.

[35] Simon J.A., Kingston R.E. (2009). Mechanisms of polycomb gene silencing: knowns and unknowns. Nat. Rev. Mol. Cell Biol..

[36] Mikkelsen T.S., Ku M., Jaffe D.B., Issac B., Lieberman E., Giannoukos G., Alvarez P., Brockman W., Kim T.K., Koche R.P. (2007). Genome-wide maps of chromatin state in pluripotent and lineage-committed cells. Nature.

[37] Gearhart M.D., Corcoran C.M., Wamstad J.A., Bardwell V.J. (2006). Polycomb group and SCF ubiquitin ligases are found in a novel BCOR complex that is recruited to BCL6 targets. Mol. Cell. Biol..

[38] Sanchez C., Sanchez I., Demmers J.A., Rodriguez P., Strouboulis J., Vidal M. (2007). Proteomics analysis of Ring1B/Rnf2 interactors identifies a novel complex with the Fbxl10/Jhdm1B histone demethylase and the Bcl6 interacting corepressor. Mol. Cell. Proteomics.

[39] Gao Z., Zhang J., Bonasio R., Strino F., Sawai A., Parisi F., Kluger Y., Reinberg D. (2012). PCGF homologs, CBX proteins, and RYBP define functionally distinct PRC1 family complexes. Mol. Cell.

[39a] He J., Shen L., Wan M., Taranova O., Wu H., Zhang Y. (2013). Kdm2b maintains murine embryonic stem cell status by recruiting PRC1 complex to CpG islands of developmental genes. Nat. Cell Biol..

[40] Carlone D.L., Skalnik D.G. (2001). CpG binding protein is crucial for early embryonic development. Mol. Cell. Biol..

[41] Carlone D.L., Lee J.H., Young S.R., Dobrota E., Butler J.S., Ruiz J., Skalnik D.G. (2005). Reduced genomic cytosine methylation and defective cellular differentiation in embryonic stem cells lacking CpG binding protein. Mol. Cell. Biol..

[42] Lee J.H., Skalnik D.G. (2005). CpG-binding protein (CXXC finger protein 1) is a component of the mammalian Set1 histone H3-Lys^4^ methyltransferase complex, the analogue of the yeast Set1/COMPASS complex. J. Biol. Chem..

[43] Lee J.H., Tate C.M., You J.S., Skalnik D.G. (2007). Identification and characterization of the human Set1B histone H3-Lys^4^ methyltransferase complex. J. Biol. Chem..

[44] Ernst P., Vakoc C.R. (2012). WRAD: enabler of the SET1-family of H3K4 methyltransferases. Briefings Funct. Genomics.

[45] Liang G., Lin J.C., Wei V., Yoo C., Cheng J.C., Nguyen C.T., Weisenberger D.J., Egger G., Takai D., Gonzales F.A., Jones P.A. (2004). Distinct localization of histone H3 acetylation and H3-K4 methylation to the transcription start sites in the human genome. Proc. Natl. Acad. Sci. U.S.A..

[46] Schneider R., Bannister A.J., Myers F.A., Thorne A.W., Crane-Robinson C., Kouzarides T. (2004). Histone H3 lysine 4 methylation patterns in higher eukaryotic genes. Nat. Cell Biol..

[47] Guenther M.G., Levine S.S., Boyer L.A., Jaenisch R., Young R.A. (2007). A chromatin landmark and transcription initiation at most promoters in human cells. Cell.

[48] Tate C.M., Lee J.H., Skalnik D.G. (2010). CXXC finger protein 1 restricts the Setd1A histone H3K4 methyltransferase complex to euchromatin. FEBS J..

[49] Blackledge N.P., Klose R. (2011). CpG island chromatin: a platform for gene regulation. Epigenetics.

[50] Wysocka J., Swigut T., Xiao H., Milne T.A., Kwon S.Y., Landry J., Kauer M., Tackett A.J., Chait B.T., Badenhorst P. (2006). A PHD finger of NURF couples histone H3 lysine 4 trimethylation with chromatin remodelling. Nature.

[51] Vermeulen M., Mulder K.W., Denissov S., Pijnappel W.W., van Schaik F.M., Varier R.A., Baltissen M.P., Stunnenberg H.G., Mann M., Timmers H.T. (2007). Selective anchoring of TFIID to nucleosomes by trimethylation of histone H3 lysine 4. Cell.

[52] Bian C., Xu C., Ruan J., Lee K.K., Burke T.L., Tempel W., Barsyte D., Li J., Wu M., Zhou B.O. (2011). Sgf29 binds histone H3K4me2/3 and is required for SAGA complex recruitment and histone H3 acetylation. EMBO J..

[53] Kim J., Daniel J., Espejo A., Lake A., Krishna M., Xia L., Zhang Y., Bedford M.T. (2006). Tudor, MBT and chromo domains gauge the degree of lysine methylation. EMBO Rep..

[54] Clouaire T., Webb S., Skene P., Illingworth R., Kerr A., Andrews R., Lee J.H., Skalnik D., Bird A. (2012). Cfp1 integrates both CpG content and gene activity for accurate H3K4me3 deposition in embryonic stem cells. Genes Dev..

[55] Eberl H.C., Spruijt C.G., Kelstrup C.D., Vermeulen M., Mann M. (2013). A map of general and specialized chromatin readers in mouse tissues generated by label-free interaction proteomics. Mol. Cell.

[56] Ruthenburg A.J., Allis C.D., Wysocka J. (2007). Methylation of lysine 4 on histone H3: intricacy of writing and reading a single epigenetic mark. Mol. Cell.

[57] FitzGerald K.T., Diaz M.O. (1999). *MLL2*: a new mammalian member of the *trx*/*MLL* family of genes. Genomics.

[58] Wang P., Lin C., Smith E.R., Guo H., Sanderson B.W., Wu M., Gogol M., Alexander T., Seidel C., Wiedemann L.M. (2009). Global analysis of H3K4 methylation defines MLL family member targets and points to a role for MLL1-mediated H3K4 methylation in the regulation of transcriptional initiation by RNA polymerase II. Mol. Cell. Biol..

[59] Birke M., Schreiner S., Garcia-Cuellar M.P., Mahr K., Titgemeyer F., Slany R.K. (2002). The MT domain of the proto-oncoprotein MLL binds to CpG-containing DNA and discriminates against methylation. Nucleic Acids Res..

[60] Bach C., Mueller D., Buhl S., Garcia-Cuellar M.P., Slany R.K. (2009). Alterations of the CxxC domain preclude oncogenic activation of mixed-lineage leukemia 2. Oncogene.

[61] Yu B.D., Hess J.L., Horning S.E., Brown G.A., Korsmeyer S.J. (1995). Altered Hox expression and segmental identity in *Mll*-mutant mice. Nature.

[62] Ernst P., Fisher J.K., Avery W., Wade S., Foy D., Korsmeyer S.J. (2004). Definitive hematopoiesis requires the mixed-lineage leukemia gene. Dev. Cell.

[63] Guenther M.G., Jenner R.G., Chevalier B., Nakamura T., Croce C.M., Canaani E., Young R.A. (2005). Global and Hox-specific roles for the MLL1 methyltransferase. Proc. Natl. Acad. Sci. U.S.A..

[64] Milne T.A., Dou Y., Martin M.E., Brock H.W., Roeder R.G., Hess J.L. (2005). MLL associates specifically with a subset of transcriptionally active target genes. Proc. Natl. Acad. Sci. U.S.A..

[65] Yokoyama A., Somervaille T.C., Smith K.S., Rozenblatt-Rosen O., Meyerson M., Cleary M.L. (2005). The menin tumor suppressor protein is an essential oncogenic cofactor for MLL-associated leukemogenesis. Cell.

[66] Scacheri P.C., Davis S., Odom D.T., Crawford G.E., Perkins S., Halawi M.J., Agarwal S.K., Marx S.J., Spiegel A.M., Meltzer P.S., Collins F.S. (2006). Genome-wide analysis of menin binding provides insights into MEN1 tumorigenesis. PLoS Genet..

[67] Zeleznik-Le N.J., Harden A.M., Rowley J.D. (1994). 11q23 translocations split the “AT-hook” cruciform DNA-binding region and the transcriptional repression domain from the activation domain of the mixed-lineage leukemia (MLL) gene. Proc. Natl. Acad. Sci. U.S.A..

[68] Chang P.Y., Hom R.A., Musselman C.A., Zhu L., Kuo A., Gozani O., Kutateladze T.G., Cleary M.L. (2010). Binding of the MLL PHD3 finger to histone H3K4me3 is required for MLL-dependent gene transcription. J. Mol. Biol..

[69] Wang Z., Song J., Milne T.A., Wang G.G., Li H., Allis C.D., Patel D.J. (2010). Pro isomerization in MLL1 PHD3-bromo cassette connects H3K4me readout to CyP33 and HDAC-mediated repression. Cell.

[70] Milne T.A., Kim J., Wang G.G., Stadler S.C., Basrur V., Whitcomb S.J., Wang Z., Ruthenburg A.J., Elenitoba-Johnson K.S., Roeder R.G., Allis C.D. (2010). Multiple interactions recruit MLL1 and MLL1 fusion proteins to the *HOXA9* locus in leukemogenesis. Mol. Cell.

[71] Park S., Osmers U., Raman G., Schwantes R.H., Diaz M.O., Bushweller J.H. (2010). The PHD3 domain of MLL acts as a CYP33-regulated switch between MLL-mediated activation and repression. Biochemistry.

[72] Muntean A.G., Tan J., Sitwala K., Huang Y., Bronstein J., Connelly J.A., Basrur V., Elenitoba-Johnson K.S., Hess J.L. (2010). The PAF complex synergizes with MLL fusion proteins at *HOX* loci to promote leukemogenesis. Cancer Cell.

[73] Meyer C., Kowarz E., Hofmann J., Renneville A., Zuna J., Trka J., Ben Abdelali R., Macintyre E., De Braekeleer E., De Braekeleer M. (2009). New insights to the MLL recombinome of acute leukemias. Leukemia.

[74] Ayton P.M., Chen E.H., Cleary M.L. (2004). Binding to nonmethylated CpG DNA is essential for target recognition, transactivation, and myeloid transformation by an MLL oncoprotein. Mol. Cell. Biol..

[75] Caslini C., Yang Z., El-Osta M., Milne T.A., Slany R.K., Hess J.L. (2007). Interaction of MLL amino terminal sequences with menin is required for transformation. Cancer Res..

[76] Muntean A.G., Hess J.L. (2012). The pathogenesis of mixed-lineage leukemia. Annu. Rev. Pathol..

[77] Mohan M., Lin C., Guest E., Shilatifard A. (2010). Licensed to elongate: a molecular mechanism for MLL-based leukaemogenesis. Nat. Rev. Cancer.

[78] Mueller D., Garcia-Cuellar M.P., Bach C., Buhl S., Maethner E., Slany R.K. (2009). Misguided transcriptional elongation causes mixed lineage leukemia. PLoS Biol..

[79] Cheung N., Chan L.C., Thompson A., Cleary M.L., So C.W. (2007). Protein arginine-methyltransferase-dependent oncogenesis. Nat. Cell Biol..

[80] Glaser S., Schaft J., Lubitz S., Vintersten K., van der Hoeven F., Tufteland K.R., Aasland R., Anastassiadis K., Ang S.L., Stewart A.F. (2006). Multiple epigenetic maintenance factors implicated by the loss of Mll2 in mouse development. Development.

[81] Austenaa L., Barozzi I., Chronowska A., Termanini A., Ostuni R., Prosperini E., Stewart A.F., Testa G., Natoli G. (2012). The histone methyltransferase Wbp7 controls macrophage function through GPI glycolipid anchor synthesis. Immunity.

[82] Andreu-Vieyra C.V., Chen R., Agno J.E., Glaser S., Anastassiadis K., Stewart A.F., Matzuk M.M. (2010). MLL2 is required in oocytes for bulk histone 3 lysine 4 trimethylation and transcriptional silencing. PLoS Biol..

[83] Glaser S., Lubitz S., Loveland K.L., Ohbo K., Robb L., Schwenk F., Seibler J., Roellig D., Kranz A., Anastassiadis K., Stewart A.F. (2009). The histone 3 lysine 4 methyltransferase, Mll2, is only required briefly in development and spermatogenesis. Epigenet. Chromatin.

[84] Chuang L.S., Ian H.I., Koh T.W., Ng H.H., Xu G., Li B.F. (1997). Human DNA–(cytosine-5) methyltransferase–PCNA complex as a target for p21^WAF1^. Science.

[85] Iida T., Suetake I., Tajima S., Morioka H., Ohta S., Obuse C., Tsurimoto T. (2002). PCNA clamp facilitates action of DNA cytosine methyltransferase 1 on hemimethylated DNA. Genes Cells.

[86] Bestor T.H., Ingram V.M. (1983). Two DNA methyltransferases from murine erythroleukemia cells: purification, sequence specificity, and mode of interaction with DNA. Proc. Natl. Acad. Sci. U.S.A..

[87] Bostick M., Kim J.K., Esteve P.O., Clark A., Pradhan S., Jacobsen S.E. (2007). UHRF1 plays a role in maintaining DNA methylation in mammalian cells. Science.

[88] Sharif J., Muto M., Takebayashi S., Suetake I., Iwamatsu A., Endo T.A., Shinga J., Mizutani-Koseki Y., Toyoda T., Okamura K. (2007). The SRA protein Np95 mediates epigenetic inheritance by recruiting Dnmt1 to methylated DNA. Nature.

[89] Avvakumov G.V., Walker J.R., Xue S., Li Y., Duan S., Bronner C., Arrowsmith C.H., Dhe-Paganon S. (2008). Structural basis for recognition of hemi-methylated DNA by the SRA domain of human UHRF1. Nature.

[90] Hashimoto H., Horton J.R., Zhang X., Bostick M., Jacobsen S.E., Cheng X. (2008). The SRA domain of UHRF1 flips 5-methylcytosine out of the DNA helix. Nature.

[91] Pradhan M., Esteve P.O., Chin H.G., Samaranayke M., Kim G.D., Pradhan S. (2008). CXXC domain of human DNMT1 is essential for enzymatic activity. Biochemistry.

[92] Bashtrykov P., Jankevicius G., Smarandache A., Jurkowska R.Z., Ragozin S., Jeltsch A. (2012). Specificity of Dnmt1 for methylation of hemimethylated CpG sites resides in its catalytic domain. Chem. Biol..

[93] Takeshita K., Suetake I., Yamashita E., Suga M., Narita H., Nakagawa A., Tajima S. (2011). Structural insight into maintenance methylation by mouse DNA methyltransferase 1 (Dnmt1). Proc. Natl. Acad. Sci. U.S.A..

[94] Syeda F., Fagan R.L., Wean M., Avvakumov G.V., Walker J.R., Xue S., Dhe-Paganon S., Brenner C. (2011). The replication focus targeting sequence (RFTS) domain is a DNA-competitive inhibitor of Dnmt1. J. Biol. Chem..

[95] Jorgensen H.F., Ben-Porath I., Bird A.P. (2004). Mbd1 is recruited to both methylated and nonmethylated CpGs via distinct DNA binding domains. Mol. Cell. Biol..

[96] Clouaire T., de Las Heras J.I., Merusi C., Stancheva I. (2010). Recruitment of MBD1 to target genes requires sequence-specific interaction of the MBD domain with methylated DNA. Nucleic Acids Res..

[97] Cross S.H., Meehan R.R., Nan X., Bird A. (1997). A component of the transcriptional repressor MeCP1 shares a motif with DNA methyltransferase and HRX proteins. Nat. Genet..

[98] Lyst M.J., Nan X., Stancheva I. (2006). Regulation of MBD1-mediated transcriptional repression by SUMO and PIAS proteins. EMBO J..

[99] Sarraf S.A., Stancheva I. (2004). Methyl-CpG binding protein MBD1 couples histone H3 methylation at lysine 9 by SETDB1 to DNA replication and chromatin assembly. Mol. Cell.

[100] Kriaucionis S., Heintz N. (2009). The nuclear DNA base 5-hydroxymethylcytosine is present in Purkinje neurons and the brain. Science.

[101] Tahiliani M., Koh K.P., Shen Y., Pastor W.A., Bandukwala H., Brudno Y., Agarwal S., Iyer L.M., Liu D.R., Aravind L., Rao A. (2009). Conversion of 5-methylcytosine to 5-hydroxymethylcytosine in mammalian DNA by MLL partner TET1. Science.

[102] Almeida R.D., Loose M., Sottile V., Matsa E., Denning C., Young L., Johnson A.D., Gering M., Ruzov A. (2012). 5-hydroxymethyl-cytosine enrichment of non-committed cells is not a universal feature of vertebrate development. Epigenetics.

[103] Almeida R.D., Sottile V., Loose M., De Sousa P.A., Johnson A.D., Ruzov A. (2012). Semi-quantitative immunohistochemical detection of 5-hydroxymethyl-cytosine reveals conservation of its tissue distribution between amphibians and mammals. Epigenetics.

[104] Inoue A., Zhang Y. (2011). Replication-dependent loss of 5-hydroxymethylcytosine in mouse preimplantation embryos. Science.

[105] Ito S., D’Alessio A.C., Taranova O.V., Hong K., Sowers L.C., Zhang Y. (2010). Role of Tet proteins in 5mC to 5hmC conversion, ES-cell self-renewal and inner cell mass specification. Nature.

[106] Ficz G., Branco M.R., Seisenberger S., Santos F., Krueger F., Hore T.A., Marques C.J., Andrews S., Reik W. (2011). Dynamic regulation of 5-hydroxymethylcytosine in mouse ES cells and during differentiation. Nature.

[107] Freudenberg J.M., Ghosh S., Lackford B.L., Yellaboina S., Zheng X., Li R., Cuddapah S., Wade P.A., Hu G., Jothi R. (2012). Acute depletion of Tet1-dependent 5-hydroxymethylcytosine levels impairs LIF/Stat3 signaling and results in loss of embryonic stem cell identity. Nucleic Acids Res..

[108] Costa Y., Ding J., Theunissen T.W., Faiola F., Hore T.A., Shliaha P.V., Fidalgo M., Saunders A., Lawrence M., Dietmann S. (2013). NANOG-dependent function of TET1 and TET2 in establishment of pluripotency. Nature.

[109] Yamaguchi S., Hong K., Liu R., Shen L., Inoue A., Diep D., Zhang K., Zhang Y. (2012). Tet1 controls meiosis by regulating meiotic gene expression. Nature.

[110] Koh K.P., Yabuuchi A., Rao S., Huang Y., Cunniff K., Nardone J., Laiho A., Tahiliani M., Sommer C.A., Mostoslavsky G. (2011). Tet1 and Tet2 regulate 5-hydroxymethylcytosine production and cell lineage specification in mouse embryonic stem cells. Cell Stem Cell.

[111] Williams K., Christensen J., Pedersen M.T., Johansen J.V., Cloos P.A., Rappsilber J., Helin K. (2011). TET1 and hydroxymethylcytosine in transcription and DNA methylation fidelity. Nature.

[112] Dawlaty M.M., Ganz K., Powell B.E., Hu Y.C., Markoulaki S., Cheng A.W., Gao Q., Kim J., Choi S.W., Page D.C., Jaenisch R. (2011). Tet1 is dispensable for maintaining pluripotency and its loss is compatible with embryonic and postnatal development. Cell Stem Cell.

[113] Wu H., Zhang Y. (2011). Tet1 and 5-hydroxymethylation: a genome-wide view in mouse embryonic stem cells. Cell Cycle.

[114] Gu T.P., Guo F., Yang H., Wu H.P., Xu G.F., Liu W., Xie Z.G., Shi L., He X., Jin S.G. (2011). The role of Tet3 DNA dioxygenase in epigenetic reprogramming by oocytes. Nature.

[115] Dawlaty M.M., Breiling A., Le T., Raddatz G., Barrasa M.I., Cheng A.W., Gao Q., Powell B.E., Li Z., Xu M. (2013). Combined deficiency of tet1 and tet2 causes epigenetic abnormalities but is compatible with postnatal development. Dev. Cell.

[116] Zhang H., Zhang X., Clark E., Mulcahey M., Huang S., Shi Y.G. (2010). TET1 is a DNA-binding protein that modulates DNA methylation and gene transcription via hydroxylation of 5-methylcytosine. Cell Res..

[117] Xu Y., Wu F., Tan L., Kong L., Xiong L., Deng J., Barbera A.J., Zheng L., Zhang H., Huang S. (2011). Genome-wide regulation of 5hmC, 5mC, and gene expression by Tet1 hydroxylase in mouse embryonic stem cells. Mol. Cell.

[118] Frauer C., Rottach A., Meilinger D., Bultmann S., Fellinger K., Hasenoder S., Wang M., Qin W., Soding J., Spada F., Leonhardt H. (2011). Different binding properties and function of CXXC zinc finger domains in Dnmt1 and Tet1. PLoS ONE.

[119] Wu H., D’Alessio A.C., Ito S., Xia K., Wang Z., Cui K., Zhao K., Sun Y.E., Zhang Y. (2011). Dual functions of Tet1 in transcriptional regulation in mouse embryonic stem cells. Nature.

[120] Wu H., D’Alessio A.C., Ito S., Wang Z., Cui K., Zhao K., Sun Y.E., Zhang Y. (2011). Genome-wide analysis of 5-hydroxymethylcytosine distribution reveals its dual function in transcriptional regulation in mouse embryonic stem cells. Genes Dev..

[121] Pastor W.A., Pape U.J., Huang Y., Henderson H.R., Lister R., Ko M., McLoughlin E.M., Brudno Y., Mahapatra S., Kapranov P. (2011). Genome-wide mapping of 5-hydroxymethylcytosine in embryonic stem cells. Nature.

[122] Iyer L.M., Tahiliani M., Rao A., Aravind L. (2009). Prediction of novel families of enzymes involved in oxidative and other complex modifications of bases in nucleic acids. Cell Cycle.

